# Absolute characterization of high numerical aperture microscope objectives utilizing a dipole scatterer

**DOI:** 10.1038/s41377-021-00663-x

**Published:** 2021-11-02

**Authors:** Jörg S. Eismann, Martin Neugebauer, Klaus Mantel, Peter Banzer

**Affiliations:** 1 Institute of Physics, University of Graz, NAWI Graz, Universitätsplatz 5, 8010 Graz, Austria; 2 Max Planck Institute for the Science of Light, Staudtstr. 2, 91058 Erlangen, Germany; 3 Institute of Optics, Information and Photonics, University Erlangen-Nuremberg, Staudtstr. 7/B2, 91058 Erlangen, Germany

**Keywords:** Imaging and sensing, Microscopy

## Abstract

Measuring the aberrations of optical systems is an essential step in the fabrication of high precision optical components. Such a characterization is usually based on comparing the device under investigation with a calibrated reference object. However, when working at the cutting-edge of technology, it is increasingly difficult to provide an even better or well-known reference device. In this manuscript we present a method for the characterization of high numerical aperture microscope objectives, functioning without the need of calibrated reference optics. The technique constitutes a nanoparticle, acting as a dipole-like scatterer, that is placed in the focal volume of the microscope objective. The light that is scattered by the particle can be measured individually and serves as the reference wave in our system. Utilizing the well-characterized scattered light as nearly perfect reference wave is the main idea behind this manuscript.

## Abstract (web summary)

An absolute characterization technique for microscope objectives is presented, working without a calibrated reference element. To achieve this, a reference wave is created by a sub-wavelength object.

## Graphical abstract

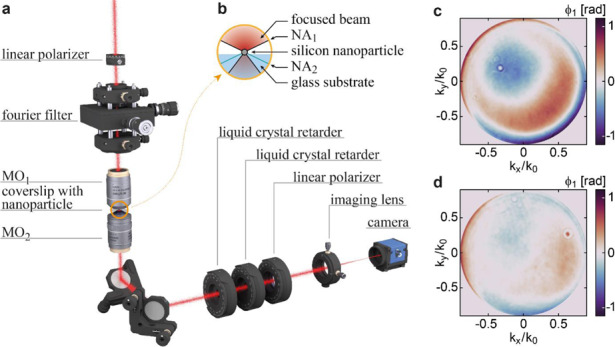

## Introduction

Measurements are something we are very familiar with. Not only in science but also in our everyday life they are ubiquitous and we perform many of them without much thought. In fact, the majority of what we designate as measurements could be equivalently called comparison. Probably the most illustrative example for this is a beam balance, where the mass of an object is determined by comparing it to known masses. There are countless other measurements involving e.g. a ruler, a measuring cup or simply a watch, that all just work because there is a calibrated device acting as a benchmark to gain the desired information. Providing such a calibrated reference can be rather challenging, especially in the realm of modern technologies and methods demanding miniaturization and increasing resolution.

In optics, a frequently occurring example is the characterization of optical elements based on the phase front of the transmitted light field. Usually, this is done by interferometry, where optical reference elements are utilized to create a reference wave. Consequently, the quality of these elements and their calibration sets an upper limit for the measurement accuracy, as their imperfections and calibration errors translate directly into the measured wavefront of the device under study. Especially when working with high numerical aperture (NA) optics, such a calibration involves its very own challenges^1,2^. Therefore, the development of so-called *absolute* characterization methods, working without a macroscopic reference object, is highly desirable.

In this work, we present an absolute characterization technique for high-NA microscope objectives. To circumvent the need for the error-prone calibration of the optical reference elements, our reference wave is created by an object smaller than the wavelength, i.e., a nanoparticle. Such a particle only supports a very limited amount of optical modes—with the dominating contributions being dipole modes^3^—which can be determined experimentally^4,5^. The main concept presented in this work is based on the utilization of the well-characterized scattering as a nearly perfect reference wave.

## Results

### Experimental scheme

For the wavefront characterization of a microscope objective (MO), or rather the experimental determination of its aberrations, it is necessary to measure the transmitted phase front. This can be achieved by either interferometric means or a specialized sensor such as a Shack−Hartmann-Sensor (SHS)^6,7^. In all cases, it is necessary to image the exit pupil (EP) of the MO under investigation (subsequently labeled as MO_1_) onto the sensor, because wave aberrations of optical elements are defined in their respective EP. We first discuss two exemplary state-of-the-art measurement techniques before presenting our own scheme.

**Fig. 1:**
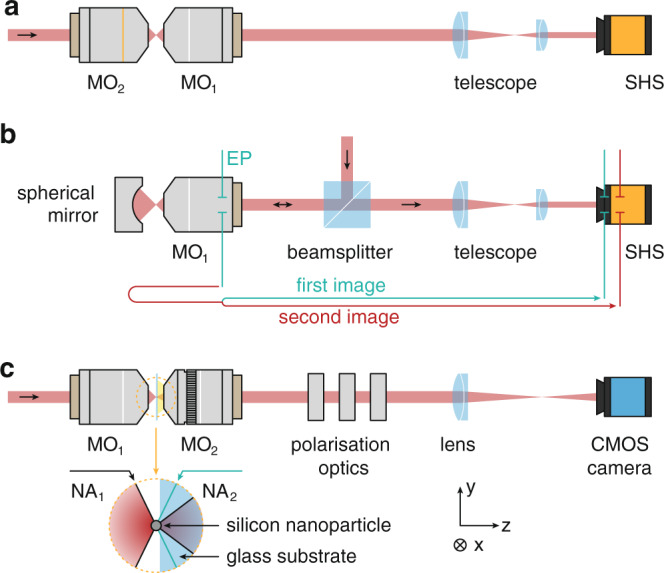
Experimental schemes for the characterization of microscope objectives (MOs). The MO under investigation is always labeled as MO_1_. **a**, **b** Conventional methods based on a Shack−Hartmann-Sensor and reference elements. In **a** MO_1_ is measured in a single pass, whereas in **b** the beam traverses MO_1_ twice. **c** Sketch of the main part of the experimental setup based on scattered light as reference wave. An incoming beam is focused and recollimated by two confocally aligned microscope objectives. A spherical silicon nanoparticle is placed on a glass substrate in the joint focal plane of the system. The back focal plane of MO_2_ (immersion type) is imaged onto a camera by an additional lens. Polarization optics, comprising two liquid crystal variable retarders and a linear polarizer, enable a polarization resolved analysis

The first scheme is depicted in Fig. 1a. There, MO_1_ is illuminated with an incoming wavefront, which after transmission carries the aberrations of MO_1_. For the illumination of MO_1_, an additional optical component, i.e., a second microscope objective MO_2_ is necessary to adapt the incoming wavefront to MO_1_. Further, a telescope is usually utilized for imaging and also for matching the size of the EP-image to the SHS. The obvious problem here is the calibration of the auxiliary optics. When removing MO_1_, the light beam coming from MO_2_ is not collimated anymore. Therefore, an additional pre-characterized MO (identical to MO_1_) acting as a benchmark is necessary to calibrate the measurement setup. Unfortunately, this would bring us back to the initial problem, namely the characterization of a microscope objective.

An alternative technique that solves the abovementioned issue is presented in Fig. 1b. There, a spherical concave mirror is used to send the light coming out of MO_1_ back, such that the beam passes the objective twice on the exact same paths, doubling the wavefront aberrations. Deviations of the concave mirror from a perfect sphere can be determined by specialized calibration procedures. The aberrations of the incoming wave as well as the beamsplitter and telescope can be determined by placing a plane mirror of known high quality to the right of MO_1_. Nonetheless, there is another source of error in this system. Seen from the SHS, the combination of MO_1_ and the reflecting surface creates two images of the EP of MO_1_, which cannot be imaged to the same plane simultaneously. The two image paths are indicated in Fig. 1b by the green and red arrow. This affects the performance of the wavefront characterization, especially if small defects need to be located precisely.

In Fig. 1c, we present an alternative and novel experimental scheme, which we describe in detail below. It was used to record all data presented in this manuscript and does not rely on any calibrated reference optics. The polarization of the incoming laser beam can be chosen arbitrarily as long as it is fixed and not changing or fluctuating with time. The beam is focused by MO_1_ and impinges onto a silicon nanoparticle, placed on a glass coverslip^8^ that is carried by a 3D piezo stage [Physik Instrumente (PI) GmbH & Co. KG, P-527 and E-710.4CL]. The size of the particle is chosen such that it predominantly supports dipole modes, with higher order modes (quadrupoles, octupoles, etc.) being strongly suppressed at the chosen wavelength^3^. The excited dipole moments are not known prior to the measurements and different microscope objectives (MO_1_) with different aberrations will lead to different combinations of dipole moments. The actual dipole moments need to be reconstructed during the measurement^5^ and their emission can then be calculated analytically^9^. The resulting dipole emission serves the purpose of a well-known, nearly perfect reference wave that can interfere with the remainder of the light, which passed through MO_1_. The transmitted beam as well as the light scattered by the particle in forward direction are collected by a confocally aligned immersion-type microscope objective (MO_2_, Leica Microsystems, HC PL FLUOTAR, ×100/1.32 OIL). The EP of both MO_1_ and MO_2_ are simultaneously imaged onto a conventional CMOS camera (The Imaging Source Europe GmbH, DMK 33UX252) by a standard achromatic lens. Using two liquid crystal variable retarders (Thorlabs Inc., LCC1113-A) and a linear polarizer in front of the imaging lens facilitates an angularly resolved full Stokes analysis^10^, which will become important during the evaluation procedure. The key feature of this setup is that it does not require any additional calibrated optical elements. After passing through MO_1_, all subsequent optical elements used for the measurement and retrieval are common-path, i.e., input beam and reference wave propagate along the exact same path. Consequently, it is not necessary to calibrate the phase aberrations of the auxiliary optics, including MO_2_. In addition, the aforementioned issue of multiple image planes for the EP does not occur in this configuration.

### Measurement

To better understand the underlying principle of our measurement strategy, we first discuss the equation for the total intensity that needs to be solved^11^:

1 $$I_{{{{\rm{tot}}}},\sigma } = I_{1,\sigma } + I_{2,\sigma } + 2\sqrt {I_{1,\sigma }I_{2,\sigma }} \cos (\phi _{1,\sigma } - \phi _{2,\sigma })$$

For a specific polarization state *σ*, this equation describes the time averaged intensity *I*_tot*,σ*_ of two interfering electromagnetic fields labeled by *i* = 1,2, where *I*_*i,σ*_ and *ϕ*_*i,σ*_ are the corresponding intensity and phase distributions, respectively. For the sake of brevity, the dependence (*k*_*x*_*, k*_*y*_) is omitted. Besides, *σ* refers to the polarization state that is selected by means of the polarization optics. In principle, the polarization state *σ* can be chosen almost arbitrarily by means of the polarization optics, as long as it is homogeneous throughout the EP. Nonetheless, it is advantageous to choose *σ* to be equal to the polarization of the input beam in order to maximize the signal-to-noise ratio. In our scenario, *i* = 1 corresponds to the incoming beam that passed MO_1_ and was transmitted into the glass substrate, carrying the aberrations of MO_1_ in its phase distribution *ϕ*_1,*σ*_. Consequently, the ultimate goal is the retrieval of *ϕ*_1,*σ*_. Strictly speaking, we are interested in the wavefront *ϕ*_1,*σ*_ without the influence of the glass substrate. However, these influences can be removed from the results in a straight-forward manner, as explained in the “Methods” section. Last, the components *I*_2,*σ*_, *ϕ*_2,*σ*_ refer to the intensity and phase distribution of the dipole wave emitted by the nanoparticle, respectively.

Without loss of generality, we choose a particle diameter of 168 nm, an input wavelength of *λ* = 680 nm and a polarization parallel to the horizontal axis (*σ* = *x*) for the experiments reported here. We characterized two MOs featuring a high numerical aperture of NA_1_ = 0.9. Due to the nature of immersion type MOs, MO_2_ can exhibit an NA significantly larger than NA_1_ (here NA_2_ = 1.32). As indicated in the lower part of Fig. 1c, this results in two distinct regions in the collected angular spectrum of MO_2_, i.e., in the recorded EP images. First, a central region $0 \le k_ \bot /k_0 \le {{{{{\mathrm{NA}}}}}}_1$, with $k_ \bot = k_x^2 + k_y^2$, where light scattered by the particle and the transmitted input beam are collected. Second, an annular region ${{{{{\mathrm{NA}}}}}}_1 \le k_ \bot /k_0 \le {{{{{\mathrm{NA}}}}}}_2$, containing only light scattered by the nanoparticle without any contribution from the transmitted input beam.

**Fig. 2:**
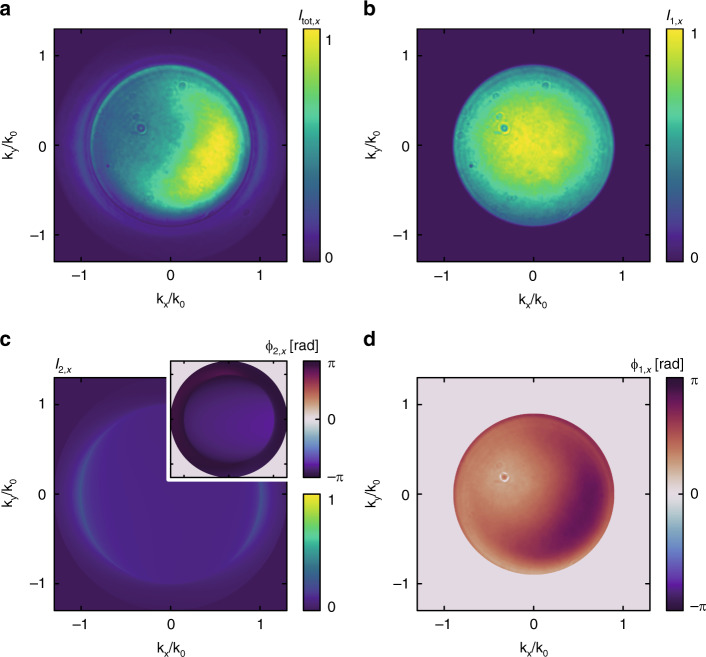
Experimental and calculated far-fields. **a**, **b** Experimental far-field images of the collection microscope objective MO_2_ with the beam focused on- and offside the particle, respectively. **c** Calculated intensity and phase distribution for the retrieved dipole moments. **d** Reconstructed phase distribution of the angular spectrum transmitted through MO_1_

The measurement procedure starts by moving the nanoparticle on the optical axis to capture the combined signal *I*_tot*,x*_ (Fig. 2a) of the excitation beam and the emission of the dipole. It is necessary to record a complete set of Stokes parameters (*x*, *y*, 45, 135, right-handed circularly polarized, left-handed circularly polarized). From this measurement, we use the aforementioned annular region above NA_1_ where only the light of the dipole wave is present to identify the underlying dipole moments. To achieve this, we calculate the far-field emission of all electric and magnetic dipoles (6 in total) placed above an interface (substrate)^9^ and use a numerical least square optimization to fit a combination of these far-fields to the measured Stokes parameters^5^. During this process, the amplitudes and phases of the dipoles serve as free parameters. The polarization resolved measurement is necessary to avoid ambiguities during this optimization. The knowledge about the induced dipole moments allows us to calculate their far-fields also in the central region of the EP, where interference with the transmitted excitation beam is observed. In other words, we extract the exact information including intensity *I*_2*,x*_ and phase *ϕ*_2,*x*_ distributions (Fig. 2c) of the reference wave, utilized for the characterization of the MO under test. A second measurement is done offside the nanoparticle with the focused beam not overlapping with the particle anymore, but only with the substrate. It enables the measurement of *I*_1,*x*_ (Fig. 2b), corresponding to a *k*-spectrum transmitted without interaction with the nanoparticle. At this point it is sufficient to record the chosen polarization state of the input beam (*x*).

With the completion of this step, all necessary variables are known to solve Eq. (1) for the desired phase distribution *ϕ*_1,*x*_. Solving such an equation generally yields two solutions. Considering the underlying physics, with *ϕ*_1_ describing the electric field that caused the excitation of a dipole described by *ϕ*_2_, we know that *ϕ*_1,*σ*_ − *ϕ*_2,*σ*_ > 0. This rules out one of the two solutions and makes the evaluation unambiguous. The corresponding result is presented in Fig. 2d.

## Discussion

### Performance analysis

**Fig. 3:**
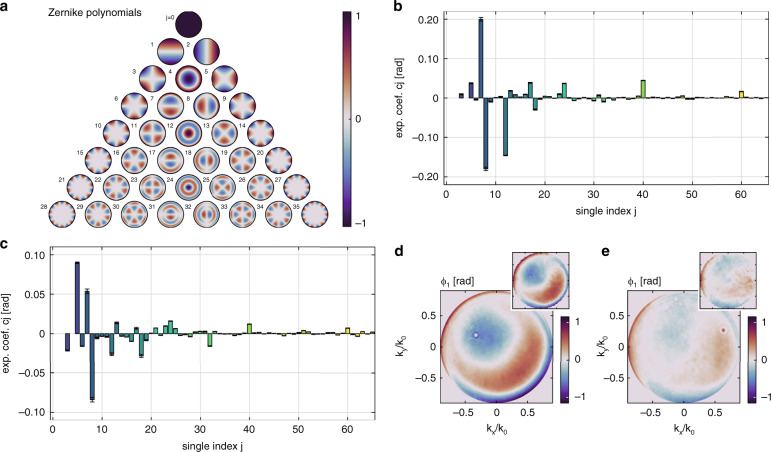
Zernike expansion. **a** Depiction of the Zernike polynomials up to *j* = 35. The associated single index *j* is indicated at the top left of each distribution. For illustrative purpose, the maximum amplitude of all polynomials is set to 1. **b**, **c** Zernike expansion coefficients of the phase distributions *ϕ*_1*,x*_ shown in **d**, **e**, respectively. Contributions from polynomials *j* = 0,1,2,4 are removed from all images. **d**, **e** Reconstructed phase distributions for two investigated MO_1_s (Leica Microsystems, HCX PL FLUOTAR, ×100/0.90 POL and Leica Microsystems, HCX PL FLUOTAR, ×100/0.90). A reference measurement recorded with SHSInspect 2Xpass by Optocraft GmbH is shown at the top right of each phase distribution

To investigate the stability and precision of our system, we perform some further analysis of the recorded data in this chapter. For this purpose, we use the so-called Zernike polynomials^12,13^, which form a continuous and orthonormal basis over a unit circle that is well suited to describe the aberrations of optical systems featuring a circular pupil. In principle, an arbitrary wavefront *ϕ*(*k*_*x*_,*k*_*y*_) can be expanded into a series of polynomials

2 $$\phi \left( {k_x,k_y} \right) = \mathop {\sum }\limits_{j = 0}^\infty c_jZ_j\left( {k_x,k_y} \right),$$

where *c*_*j*_ denotes the expansion coefficients and *Z*_*j*_ are actual Zernike polynomials in the single index representation^13^. We show the distributions of *Z*_*j*_ up to *j* = 35 in Fig. 3a.

We now use Eq. (2) to decompose the experimentally retrieved phase distribution *ϕ*_1,*x*_ into Zernike polynomials. For our further analysis we set the expansion limit to *j* = 65. Figure 3b, c shows the expansion coefficients for the two previously mentioned MO_1_s that were tested. The coefficients for *j* = 0,1,2,4 are removed from the images for the following reasons: *Z*_0_, called piston or bias, is only a constant phase offset. *Z*_1_*, Z*_2_ (tip, tilt) describe phase ramps along *k*_*x*_ and *k*_*y*_, respectively, that could be compensated by simply tilting MO_1_. *Z*_4_ refers to a first-order defocus that can be corrected by moving MO_1_ along the optical axis (*z*). Being heavily influenced by the physical alignment, these four contributions are not only of less importance, but they also yield results with rather high fluctuations when repeating the characterization.

In Fig. 3d, e we present the final experimentally retrieved phase distributions with the aforementioned four contributions removed. The coefficients shown in Fig. 3b, c correspond to the distribution Fig. 3d, e, respectively. The data presented in Fig. 3b, d correspond to the same MO_1_ that was shown already in Fig. 2. In addition, we show a reference measurement at the top right of both reconstructed phase distributions. These measurements were recorded by Optocraft GmbH with their SHSInspect metrology platform in the 2Xpass configuration. This system is based on the principle that was shown in Fig. 1b. As can be seen, our results show excellent agreement with the independently recorded reference dataset.

To showcase the performance of our method, several additional checks were done that are explained in more detail in the “Methods” section. The results and error bars shown in Fig. 3 are retrieved by averaging 30 measurements for each of the two microscope objectives and highlight already the outstanding precision of the system.

### Conclusion and outlook

In summary, we have developed and demonstrated an absolute method for the characterization of high numerical aperture microscope objectives by using a dipole scatterer in order to create a well-known reference wave. When performing microscopy of almost any kind, the microscope objective is without doubt the key element to determine both the resolution and the quality of the created images. This renders our presented method highly relevant for the development of cutting-edge microscopy systems but also for all kinds of experimental setups where a microscope objective is involved. Working with a characterized microscope objective and knowing its errors enables the implementation of error correction strategies and allows for quantitative measurements.

In general, the method is rather flexible in terms of what microscope objectives can be used, but there are some restrictions that need to be satisfied. First of all, NA_2_ > NA_1_, to get access to an outer region in the recorded BFP images that is used to reconstruct the excited dipole moments. In the current configuration optimized for the characterization of dry MOs, this is not an issue due to NA_1_ being theoretically capped at 1. Second, although there is no strict limit for how low NA_1_ can be, a lower NA_1_ generally results in larger foci and therefore less scattering for a fixed particle size. Consequently, a lower NA_1_ will increase the errors of the measurements. However, low-NA optics usually do not require such precise characterization of the transmitted phase front and there are many existing methods that are sufficient for these components. In principle, the scheme could also be used to characterize immersion-type MOs, as long as the scattering particle still behaves like a dipolar scatterer with negligible contributions of higher order multipoles when embedded in oil. In some cases, a different strategy will be required to identify the excited dipole moments as it is not always possible to choose NA_2_ > NA_1_ anymore. Powerful solutions for this could be cross polarization or structured illumination, but these go beyond the scope of this manuscript.

It should be noted here that this technique is not restricted to the chosen wavelength. Although for a fixed nanostructure the potential spectral range is limited, the complete visible and near infrared spectral range can be covered by using particles of other sizes or materials. Furthermore, it is also not necessary to use a perfectly spherical nanostructure, since our procedure is capable of identifying arbitrary combinations of dipoles. As long as they feature a reasonably strong dipole response and simultaneously suppress higher order multipoles, it is actually possible to use almost arbitrarily shaped nanostructures. However, tailoring the size and shape of the particle can also offer a promising route to improve the precision of our technique even further. The main goal here is to minimize the amount of modes supported by the particle to still enable a simple data analysis. Any higher order mode that is not considered in the determination of the scattered light will contribute to a phase error in the reconstructed phase front. But also, the considered modes are measured with a limited accuracy, which leads to measurement errors. Accordingly, promising particle shapes for improving our approach are flat cylindrical particles, supporting mainly three dipole modes^14,15^, or nanorods that predominantly support only a single dipole mode^16^. In particular, metal cylinders (e.g. made from gold etc.) would be the most promising alternative to the spherical nanoparticles used in this work. Using modern lithography or milling techniques, cylindrical nanoparticles can be fabricated easily in arrays including different sizes, hence providing a full range of different probes on a single sample to cover and measure over a wide spectral range. Further, the method can be extended with ease to also detect the sensitivity of a microscope objective to different input polarization states, allowing for a detailed analysis of the birefringence of the MO. All necessary polarization optics for this extended analysis are already present as they are required for the detection of the dipole moments. Our experimental approach offers a powerful, versatile and novel method for the characterization of high-NA optics, which are used in the majority of microscopy, imaging, and sensing devices.

## Materials and methods

### Positioning of the nanostructure

For a part of the measurement, it is necessary to place the nanostructure at the focal spot of the system. To find out where the nanoparticle is roughly located, the two confocally aligned MOs and the camera can be used as a scanning microscope. For this purpose, the sample is raster scanned through the focal volume and the intensity on the camera is integrated. The nanostructure then becomes visible as a dip in the integrated transmitted intensity distribution. Once the nanoparticle is roughly positioned in the beam, there are several ways how the fine positioning can be achieved. One possibility is to perform a finer scan around the position of the scatterer, followed by a center of mass calculation to find out where the minimum in the distribution of the transmitted light is. It is also feasible to use the distribution of the scattered light in the annular region of the EP above NA_1_ to retrieve the relative position between the focused beam and the particle^17^. Both procedures easily achieve a precision below 10 nm, which is better than required as will become clear in the error analysis below.

### Influence of the glass substrate

During the measurements, the air−glass boundary is very close to the focal plane, where the beam diameter is below 1 µm. The surface unevenness across such a small area is negligible. Further, traversing from air to glass introduces a defocus and spherical aberrations as additional wave aberrations. In order to calculate these aberrations, it is necessary to know the position of the interface relative to the focused beam. Assuming an aberration-free MO_1_, this position can be determined from the measured data. Since the actual wave aberrations of MO_1_ would only give rise to higher order corrections to the positions of both particle and interface, which are negligible, this assumption is justified. Then, the additional spherical aberration introduced by the substrate interface can be determined and subtracted from the measurement results.

### Error analysis

Several tests were performed to examine the reliability of the proposed method. To quantify the similarity between two measurements, we proceed as follows. First, the contributions of the Zernike polynomials *Z*_*j*_ for *j* = 0,1,2,4 are calculated and subtracted from the individual reconstructed phase distributions. Thereafter, we compute the root-mean-square of the difference of the two corrected phase distributions. Last, the results are expressed in units of the wavelength *λ*. All tests were repeated several times, as noted. Further, no identical test was performed twice in a row. There was always at least one of the other tests performed in between.

#### Repeatability

The measurement is performed twice consecutively. The test was done eight times. The average measured deviation is *λ*/2705.

#### Reproducibility

The measurement is performed twice where in between two measurements the experimental setup is realigned. More specifically, after the first measurement, the particle is moved out of the focused beam and MO_1_ was moved transversely to a position where no light was collected by MO_2_ anymore. Afterwards, MO_1_ is realigned, the particle is brought back to the center of the beam and the second measurement is performed. The test was conducted four times. The average measured deviation is *λ*/456.

#### Systematic particle movement

In order to investigate how critical the positioning of the particle is, the scatterer was intentionally displaced by a distance ±*D* along the *x*- or *y*-axis. The measurements at the two positions are then compared to a third measurement at the center. The test was done four times. For values of *D* = {20, 40, 60, 80, 100} nm, the resulting deviations are *λ*/{1515, 773, 539, 377, 277}. The results clearly show that larger misplacements of the nanoparticle away from the optical axis lead to increasing deviations. Most likely, this is due to the phase ramp that is imprinted to the dipole wave once its origin is not on the optical axis anymore. The associated errors occur dominantly at the edge of the aperture of MO_1_ where such a phase ramp can quickly result in a relative phase between the two interfering components that exceeds 2*π*. Such a large phase difference would require additional care in the evaluation algorithm. However, as the position of the particle can be comfortably aligned with a precision below 10 nm, this problem does not necessarily need to be solved. Displacements up to 60 nm still result in deviations smaller than the reproducibility values of the system.

## Data Availability

Data underlying the study are available from the authors upon request.
